# Co-occurrence subgroups of child sexual abuse, health risk behaviors and their associations among secondary school students in China

**DOI:** 10.1186/s12889-021-11199-5

**Published:** 2021-06-14

**Authors:** Yan Zhang, Xiayun Zuo, Yanyan Mao, Qiguo Lian, Shan Luo, Shucheng Zhang, Xiaowen Tu, Chaohua Lou, Weijin Zhou

**Affiliations:** 1 School of Public Health, Fudan University, 779 Laohumin Road, Shanghai, 200237 China; 2 NHC Key Lab. of Reproduction Regulation (Shanghai Institute of Planned Parenthood Research), School of Public Health, Fudan University, 779 Laohumin Road, Shanghai, 200237 China; 3 West China Second University Hospital, Sichuan University, Chengdu, Sichuan China; 4 National Research Institute for Family Planning, Beijing, China

**Keywords:** Middle school students, Child sexual abuse, Health risk behaviors, Latent class analysis

## Abstract

**Background:**

Little is known on the co-occurrence and heterogeneity of child sexual abuse (CSA) or health risk behavior (HRB) prevalence nor the associations among the victims.

**Objectives:**

To detect the prevalence and subgroups of adolescents reporting CSAs or HRBs, and to examine the association between the subgroups.

**Methods:**

Participants were secondary school students in a national survey in China (*N* = 8746). Self-reported CSA and HRB experiences were collected through a computer assisted questionnaire. Prevalence and confidence intervals were calculated. Multigroup latent class analysis (LCA) was used to examine latent subgroups of CSA and HRB. Dual latent class regression analysis was used to examine the association between CSA and HRB classes.

**Results:**

A total of 8746 students participated in our study. The prevalence of having ever experienced any of the reported seven CSA items was 12.9%. The preferred LCA model consisted of a three-class CSA latent variable, i.e. “Low CSAs”(95.7% of the total respondents), “Verbal or exhibitionism CSAs”(3.3%), and “high multiple CSAs” (1.1%); and a three-class HRB latent variable, i.e. “Low HRBs”(70.5%), “externalizing HRBs” (20.7%), and “internalizing HRBs” (8.7%). Students in the “Verbal or exhibitionism CSAs” or “high multiple CSAs” classes had higher probabilities of being in “externalizing HRBs” or “internalizing HRBs” classes. The probabilities were higher in “high multiple CSAs” class(male externalizing OR 4.05, 95%CI 1.71–9.57; internalizing OR 11.77, 95%CI 4.76–29.13; female externalizing OR 4.97, 95%CI 1.99–12.44; internalizing OR 9.87, 95%CI 3.71–26.25) than those in “Verbal or exhibitionism CSA”(male externalizing OR 2.51, 95%CI 1.50–4.20; internalizing OR 3.08, 95%CI 1.48–6.40; female externalizing OR 2.53, 95%CI 1.63–3.95; internalizing OR 6.05, 95%CI 3.73–9.80).

**Conclusions:**

Prevalence of CSA items varies. Non-contact CSAs are the most common forms of child sexual abuse among Chinese school students. There are different latent class co-occurrence patterns of CSA items or HRB items among the respondents. CSA experiences are in association with HRB experiences and the associations between latent classes are dose-responded. Multi-victimization has more significantly negative effects. The results could help identify high-risk subgroups and promote more nuanced interventions addressing adverse experiences and risk behaviors among at-risk adolescents.

**Supplementary Information:**

The online version contains supplementary material available at 10.1186/s12889-021-11199-5.

## Background

Child Sexual Abuse (CSA) means the involvement of a child in sexual activity that he or she does not fully comprehend, is unable to give informed consent to, or for which the child is not developmentally prepared and cannot give consent, or that violates the laws or social taboos of society [1]. CSA has spread throughout countries, continents, and socioeconomic classes in recent decades [2] and attracted increasing attention from the public, the media, and the academia. WHO [3] estimated in 2006 that 150 million girls and boys under the age of 18 have experienced CSA. Some meta-analyses estimated the average global prevalence of CSA to be around 11.8%, although it varies from 2 to 62% among different studies as well as types of CSA [4–6].

CSA is regarded as a type of Adverse Childhood Experiences. Studies, particularly among youth, have shown CSA victims are more likely to engage in behaviors that can place them at risk for negative outcomes, which are in general referred to as youth Health Risk Behavior (HRB), such as alcoholism, drug abuse, anxiety, violence, and suicidal ideation [7, 8], and has long been concerned with short-term and long-term negative sequelae [9–16]. Even a single form of abuse can be associated with risk in different HRB domains [17–19]. CSA is however a multi-indicator measurement, which indicates that experiencing CSA often means experiencing more than one form of CSA [20, 21] i.e. multiple victimization [22, 23]. While the consequence of CSA may not be measurably apparent for some youth, other youth report HRBs}{suicidal ideation [24, 25]. Different forms of abuse items may overlap and interact with each other, and the combination of different forms may modify specific social and psychological consequences [23, 26, 27]. CSA’s covert nature may prevent victims from expressing themselves and seeking help. Without considering the nature of co-occurrence, the estimation of the association between CSA and HRB may be biased [28]. The lack of research on multi-victimization hinders our understanding of the impact of multiple CSA victimization [29]. Insights into different subgroups of CSA and the association patterns with HRBs could help service providers and decision makers identify adolescents at risk that need help, and design specific inventions according to victims’ characteristics and demands [30–32].

China has the largest youth population in the world, with more than 200 million students in primary and secondary schools, accounting for 20% of the national population in total. CSA has attracted public attention in China, while researches in this field are still insufficient, with scarcely any on multiple victimization. Existing Chinese researches on the association between child victimization and health behaviors often focused on single form of the adolescent adverse experiences from small samples or limited study sites [33–35]. It is thought that the cultures and traditions of Asian societies may lead to different CSA patterns and their associations with behaviors, but there is little research on CSA patterns [36] in China. There is need to examine the heterogeneity and patterns of multi-victimization in teens to help optimize prevention and intervention programs on a national scale.

## Methods

### Study aims

This study aimed to investigate the prevalence of CSA and HRB along with the latent co-occurrence patterns, and to test the hypothesis that the subtypes of CSA are associated with HRB sub-types.

### Survey design, sites and population

This was a cross-sectional school-based study. The data for the analysis came from a national reproductive and health survey of secondary school students in China. Given China’s population diversity, multistage sampling was applied to recruit study participants. Seven provinces/autonomous regions (Shandong, Guangxi, Hebei, Heilongjiang, Sichuan, Shaanxi, Inner Mongolia) representing geographic or social variation were selected. Then, one city/town with a moderate level of economic development was selected in each province/autonomous region. In each city/town, one urban area and one rural area were selected, after which one junior secondary school and one senior secondary school were selected in each area. Two classes in each grade (including all three grades in junior secondary school and all three grades in senior secondary school) were invited to participate in the survey using cluster random sampling.

### Survey implementation and data collection

This survey was conducted between year 2014 and 2015. Data collection was through a questionnaire with a computer-assisted self-interviewing (CASI) approach. The content of the questionnaire related to this analysis included a set of designed questions on experiences of CSA items, experiences of HRB items, and some covariates. During the survey day, each student took a separate seat in the school’s computer room and completed the electronic questionnaire. The questionnaire was anonymous, and the subjects could not obtain information of others even using the same computer afterwards. Field investigators assisted the students by monitoring or providing explanations when necessary to avoid ambiguity, however, they could not see or interfere with the respondent’s answers. If any respondent feels uncomfortable with any question, he or she can skip part or all of the content. The data generated after the investigation were uniformly stored and processed by the researchers after deprivacy.

### CSA scale designing and testing

CSA refers to reluctant sexually related experiences encountered by the participated students aged 10-18 years. Sexual abuse experiences from peers were excluded. Due to the length of the investigation, we modified the ISPCAN Child Abuse Screening Tool (ICAST-R) [37] and Chen’s instrument [38] to obtain a set of seven-item self-reported CSA scale before the age of 18. Chen’s 12-item CSA questionnaire is the most widely used CSA scale in Chinese CSA study [39]. Compared with Chen’s instrument, our CSA measurements made these following adjustments: adding the question “Having been told dirty jokes or shown pornographic pictures, publications or supplies, etc” as one form of non-physical contact CSA, deleting the question “Tried to sexually arouse the child” and “Made child arouse them and touch their body in a sexual way” because they were hard to understand for children, deleting “Tried to have anal intercourse with child” and “Had anal intercourse with child” due to its abstruseness and the rare prevalence reported, combining “Exposed their genitals to the child” and “Masturbated in front of the child” into one question” Having seen someone exposing his/her genitals or masturbating in front of you”, combining “Touched child’s genitals with their mouth” and “Made child touch their genitals with child’s mouth” into one question” Having had someone touching your privates/breasts or forcing you to touch his/her privates/breasts”. Our seven-item two-category (yes/no) self-reported CSA scale has a Cronbach’s alpha 0.65 [37–39].

### Measures

Child sexual abuse (CSA): The study includes a set of seven-item two-category (yes/no) self-reported CSA scale (Table 1).

**Table 1:** Definitions of CSA and HRB items

		Response
**Child sexual abuse items (CSAs)**		
CSA1	Have you ever had been told dirty jokes or shown pornographic pictures, publications or supplies, etc.?	0 = No, 1 = Yes
CSA2	Have you ever had seen someone exposing his/her genitals or masturbating in front of you?	0 = No, 1 = Yes
CSA3	Have someone ever touched your privates/breasts or forcing you to touch his/her privates/breasts?	0 = No, 1 = Yes
CSA4	Have someone ever touched rubbed his/her genitals on you?	0 = No, 1 = Yes
CSA5	Have someone ever touched your genitals or forcing you to make contact with his/her genitals by mouth?	0 = No, 1 = Yes
CSA6	Have someone ever touched attempted to have sex with you?	0 = No, 1 = Yes
CSA7	Have someone ever touched forced to have sex with you?	0 = No, 1 = Yes
**Health risk behaviors items (HRBs)**		
HRB01	Have you ever had sexually intimate behaviors such as hugging, kissing, touching breasts, genitals, thighs, or having intercourse?	0 = No, 1 = Yes
HRB02	Have you ever smoking (including even smoked just a cigarette or two)?	0 = No, 1 = Yes
HRB03	Have you ever had a habit of drinking alcohol (refers to drinking alcohol at least once a month, including beer, liquor, wine, etc.)?	0 = No, 1 = Yes
HRB04	Have you ever been depressed, feeling despair, or extremely anxious for over two weeks or more?	0 = No, 1 = Yes
HRB05	Have you ever seriously considered committing suicide?	0 = No, 1 = Yes
HRB06	Have you ever committed suicide?	0 = No, 1 = Yes
HRB07	Have you gambled in the past year(not including activities such as playing mahjong or porker with friends or relatives for entertainment purposes and for winning a small amount of money or jackpot)?	0 = No, 1 = Yes
HRB08	Have you fought with acquaintances / classmates / strangers in the past year?	0 = No, 1 = Yes
HRB09	Have you skipped classes during the last year?	0 = No, 1 = Yes
HRB10	Have you ever run away from home?	0 = No, 1 = Yes

Health risk behaviors (HRB): The study includes several risk behaviors that cover different behavioral dimensions. These questions were transformed into a set of two-category items (yes / no) (Table 1). The HRB scale has a Cronbach’s alpha 0.69.

### Covariates

Covariates in this analysis were demographic characteristics including the respondents’ residential area, age, school grade, whether to live on campus, peer relationship, overall feeling of school, academic performance, the respondents’ free time lifestyle such as reading/playing video games/chatting/regular physical exercise/internet surfing, and the respondents family characteristics such as number of siblings, family economic condition, overall feeling of family, parent’s education, relationship with parents, parents disciplinary (Table 2).

**Table 2:** Participant demographic characteristics

	Full Sample	Male	Female
	n (%)	n (%)	n (%)
Area			
Urban	4305 (49.2)	2168 (50.0)	2137 (48.5)
Rural	4441 (50.8)	2171 (50.0)	2270 (51.5)
Grade			
Seventh grade	2246 (25.7)	1191 (27.4)	1055 (23.9)
Eighth grade	1147 (13.1)	625 (14.4)	522 (11.8)
Ninth grade	1021 (11.7)	517 (11.9)	504 (11.4)
Tenth grade	2431 (27.8)	1133 (26.1)	1298 (29.5)
Eleventh grade	1092 (12.5)	483 (11.1)	609 (13.8)
Twelfth grade	809 (9.2)	390 (9.0)	419 (9.5)
Age			
12 y	350 (4.0)	153 (3.5)	197 (4.5)
13 y	1756 (20.1)	895 (20.6)	861 (19.5)
14 y	1207 (13.8)	687 (15.8)	520 (11.8)
15 y	1101 (12.6)	562 (13.0)	539 (12.2)
16 y	1418 (16.2)	655 (15.1)	763 (17.3)
17 y	1574 (18.0)	732 (16.9)	842 (19.1)
18 y	1340 (15.3)	655 (15.1)	685 (15.5)
Whether to live on campus			
No	4404 (50.4)	2349 (54.1)	2055 (46.6)
Yes	4342 (49.6)	1990 (45.9)	2352 (53.4)
Academic performance			
Good	3605 (41.2)	1705 (39.3)	1900 (43.1)
Fair	3602 (41.2)	1686 (38.9)	1916 (43.5)
Poor	1539 (17.6)	948 (21.8)	591 (13.4)
Peer relationship			
Good	5255 (60.1)	2610 (60.2)	2645 (60.0)
Fair	3266 (37.3)	1605 (37.0)	1661 (37.7)
Poor	225 (2.6)	124 (2.9)	101 (2.3)
Sibling number			
None	4350 (49.7)	2436 (56.1)	1914 (43.4)
One	3165 (36.2)	1385 (31.9)	1780 (40.4)
Two or more	1231 (14.1)	518 (11.9)	713 (16.2)
Marital status of parents			
Normal / married	7782 (89.6)	3866 (89.9)	3916 (89.3)
Divorced / separation	670 (7.7)	326 (7.6)	344 (7.8)
Death of one or both parents	233 (2.7)	110 (2.6)	123 (2.8)
Parents’ relationship			
Harmonious	5189 (66.7)	2564 (66.3)	2625 (67.0)
Not harmonious	2593 (33.3)	1302 (33.7)	1291 (33.0)
Father’s education			
Elementary and below	1298 (15.7)	603 (14.8)	695 (16.6)
Junior high school	4276 (51.9)	2050 (50.5)	2226 (53.2)
High school / Vocational school	2002 (24.3)	1038 (25.5)	964 (23.0)
College and above	670 (8.1)	372 (9.2)	298 (7.1)
Relationship with father			
Good	5117 (58.5)	2517 (58.0)	2600 (59.0)
Fair	2283 (26.1)	1153 (26.6)	1130 (25.6)
Poor	1346 (15.4)	669 (15.4)	677 (15.4)
Severity of father			
Strict	1199 (13.7)	626 (14.4)	573 (13.0)
Average	6510 (74.4)	3208 (73.9)	3302(74.9)
Easy-going	1037 (11.9)	505 (11.6)	532 (12.1)
Mother degree			
Elementary and below	1244 (14.2)	572 (13.2)	672 (15.2)
Junior high school	4092 (46.8)	1948 (44.9)	2144 (48.6)
High school / Vocational school	1810 (20.7)	935 (21.5)	875 (19.9)
College and above	1600 (18.3)	884 (20.4)	716 (16.2)
Relationship with mother			
Good	5926 (67.8)	2914 (67.2)	3012 (68.3)
Fair	1949 (22.3)	1014 (23.4)	935 (21.2)
Poor	871 (10.0)	411 (9.5)	460 (10.4)
Severity of mother			
Strict	1771 (20.2)	881 (20.3)	890 (20.2)
Average	6370(72.8)	3146(72.5)	3224(73.2)
Easy-going	605 (6.9)	312 (7.2)	293 (6.6)
free time lifestyle			
Staying at home	3702 (42.3)	1494 (34.4)	2208 (50.1)
Watching TV/video	4089 (46.8)	1643 (37.9)	2446 (55.5)
sports	2074 (23.7)	1345 (31.0)	729 (16.5)
Reading	2586 (29.6)	1098 (25.3)	1488 (33.8)
Internet surfing	6264 (71.6)	3267 (75.3)	2997 (68.0)
**Total**	**8746(100)**	**4339(100)**	**4407(100)**

### Ethics

The research has been reviewed and approved by the local ethics committee. Before the survey was conducted, the purpose and implementation of the study were explained to the students, teaching staff, and parents in the parents meeting whose children were on the invitation list of the sample classes. Anonymity and confidentiality were guaranteed and the rights to refuse or terminate participation in the investigation were understood. Students and parents who were willing to accept the invitation to the study were asked and signed a written consent form.

### Power calculation

Based on the sampling framework, about 50 students were planned to investigate of each sex and each age group from each site and the expected sample size was 8400. All students attended to school in the sample classes on the survey day were invited to our survey. Finally, we yielded a total sample of 8746 students. In this study, the associations between CSAs and HRBs were analyzed. According to the literature [32], the OR between CSAs and HRBs, such as depression, suicidal ideation and substance abuse, etc., was between 1.2 and 2.9. Considering 2 as the estimated OR value, we conducted the power estimation based on the total sample size of 8746 cases, and then the statistical power(1-β) could reach 82.7%.

### Analytic approach

Descriptive statistics were conducted to examine the prevalence of CSA items and HRB items. Categorical data were reported in count and percentage. Then a multi-group latent class analysis (LCA) was adopted to detect profiles of CSA and profiles of HRB across genders to explain the relation between several categorical manifest variables (indicators) by one or more underlying latent categories (classes). Model fit statistics such as the Bayesian information criterion (BIC), the Akaike information criterion (AIC), and the interpretability were considered when evaluating model fit. Once the preferred LCA models were conducted, we got two latent categorical variables (CSA profile and HRB profile). We determined the most likely latent class membership using the highest posterior class probability for each individual. To explore the association of CSA profile and HRB profile, dual LCA regression stratified by gender was used to explore the association of the two latent categorical variables. Covariates can be incorporated in the dual LCA regression model using a logistic link function. Missing data were replaced using full information maximum likelihood (FIML) estimation, which is the default in Mplus. Sawtooth Software was used to classify data from the questionnaire. Data was checked and cleaned site by site, and then merged and exported into Stata format via Stata version 15.1. Descriptive analysis was conducted in R version 3.6.1. Latent class analysis and the dual LCA regression were completed using Mplus version 7.4 [40].

## Results

### Participant demographics

According to the list of survey respondents, 8910 students were consented to take part in the survey and supposed to be at school on the date of survey. However, 164 were absent due to sickness or personal affairs on the actual survey date. As a result, the total number of respondents included in the survey were 8746, with a response rate of 98.2%. Ages of the participants ranged from 12 to 18 years, with a mean of 15.37 ± 1.95. The number of male students participated in the survey was 4339(49.6%), and that of female students was 4407(50.4%). Proportions of other characteristics were shown in Table 2.

### Prevalence of CSA and HRB items

The prevalence of the CSA indicators is shown in the upper half of Table 3. In general, a total of 1131 respondents reported to have had at least one CSA experience, with an overall proportion of 12.9%. Among all respondents, 527 (6.0%) had experienced non-physical contact CSA, while 357 (4.1%) had experienced physical-contact CSA with 247(2.8%) having had experienced attempted intercourse or forced intercourse. Between the genders, non-physical contact CSA and physical-contact CSA were more commonly experienced while attempted intercourse or forced intercourse were less seen in males. The prevalence of CSA stratified by covariates such as individual, familial or school factors could be seen in Additional Table 1.

**Table 3:** Prevalence of CSA and HRB by gender and type of abuse **(N (%))**

	Full Sample (*N* = 8746)	Male (*N* = 4339)	Female (*N* = 4407)
**Child sexual abuse items (CSAs)**			
**Having experienced at least one CSA**	**1131 (12.9)**	**613 (14.1)**	**518 (11.8)**
**Non-physical contact CSA**	**527 (6.0)**	**296 (6.8)**	**231 (5.2)**
CSA1: Having been told dirty jokes or shown pornographic pictures, publications or supplies, etc.	382 (4.4)	234 (5.4)	148 (3.4)
CSA2: Having seen someone exposing his/her genitals or masturbating in front of you	389 (4.4)	208 (4.8)	181 (4.1)
**Physical-contact CSA**	**357 (4.1)**	**211 (4.9)**	**146 (3.3)**
CSA3: Having had someone touching your privates/breasts or forcing you to touch his/her privates/breasts	352 (4.0)	184 (4.2)	168 (3.8)
CSA4: Having had someone rubbing his/her genitals on you	166 (1.9)	100 (2.3)	66 (1.5)
CSA5: Having had someone touching your genitals or forcing you to make contact with his/her genitals by mouth	102 (1.2)	64 (1.5)	38 (0.9)
**Attempting intercourse/intercourse**	**247 (2.8)**	**106 (2.4)**	**141 (3.2)**
CSA6: Having had someone attempting to have sex with you	211 (2.4)	88 (2.0)	123 (2.8)
CSA7: Having had someone forcing to have sex with you	85 (1.0)	39 (0.9)	46 (1.0)
**Health risk behaviors items (HRBs)**			
**Having had at least one risk behavior**	**3450 (39.4)**	**1149 (26.5)**	**2301 (52.2)**
HRB01: Sex-related behavior	658 (7.5)	486 (11.2)	172 (3.9)
HRB02: CSA: Smoking	1877 (21.5)	1491 (34.4)	386 (8.8)
HRB03: Drinking alcohol	1893 (21.6)	1289 (29.7)	604 (13.7)
HRB04: Depression and anxiety	1952 (22.3)	985 (22.7)	967 (21.9)
HRB05: Suicidal ideation	1069 (12.2)	448 (10.3)	621 (14.1)
HRB06: Suicidal behavior	223 (2.5)	84 (1.9)	139 (3.2)
HRB07: Gambling	532 (6.1)	394 (9.1)	138 (3.1)
HRB08: Skipping classes	964 (11.0)	619 (14.3)	345 (7.8)
HRB09: Fighting	743 (8.5)	437 (10.1)	306 (6.9)
HRB10: Running away from home	2300 (26.3)	1698 (39.1)	602 (13.7)

The prevalence of the HRB indicators are shown in the lower part of Table 3. There were 39.4% that had at least one risk behavior. For males, there was a higher prevalence of “sex-related behavior”, “smoking”, “drinking alcohol”, “gambling”, “skipping classes “, “fighting” and “running away from home”. For females, there was a higher prevalence of having a suicidal ideation, (female 14.1% vs. male 10.3%) or suicidal behavior (female 3.2% vs. male 1.9%).

### LCA model selection

Table 4 shows the fit statistics of the latent class analysis models for CSAs and HRBs. To select a suitable latent class model for the seven CSA items, multi-group LCA models were estimated from two to nine latent classes. As can be seen in Table 4, the three-class solution CSA latent class had the lowest BIC value (25,544.357), and the lowest AIC value (25,214.351). As for HRB, the three-class solution has the lowest BIC (71,091.7). Although the AIC value at this point was not the smallest, there was an obvious turning point to show a steep drop before the point and a slow descent after the point. Meanwhile, the three-class model has a clear, meaningful interpretation. The probabilities of classification error of both models were fairly accepted (CSA 0.0369, HRB 0.1314). Therefore, this analysis considered the three-classification model as the most suitable latent class model for CSAs and HRBs.

**Table 4:** Parameters for LCA model selection

	AIC	BIC	aBIC	Entropy	Classification Error
CSA models					
2-class model	25,244.969	26,129.973	25,449.022	0.981	0.0089
3-class model	25,214.351	25,544.357	25,420.402	0.907	0.0369
4-class model	25,217.654	25,563.405	25,451.566	0.96	0.0516
5-class model	25,230.403	25,642.235	25,506.741	0.955	0.0611
6-class model	25,237.068	25,745.078	25,581.271	0.905	0.0664
7-class model	25,280.811	25,867.275	25,646.413	0.907	0.0915
8-class model	25,271.005	25,980.084	25,748.634	0.872	0.1034
9-class model	25,332.067	26,226.312	25,797.306	0.88	0.2096
HRB models					
2-class model	70,809.482	71,255.292	71,055.089	0.842	0.0903
3-class model	70,497.285	71,091.699	70,824.761	0.837	0.1033
4-class model	70,410.626	71,153.643	70,819.971	0.791	0.1314
5-class model	70,320.915	71,212.535	70,812.129	0.776	0.1518
6-class model	70,333.206	71,373.43	70,906.289	0.746	0.2151
7-class model	70,305.65	71,494.477	70,960.602	0.735	0.2453
8-class model	70,296.554	71,633.984	71,033.375	0.749	0.2854
9-class model	71,587.223	71,884.43	71,750.962	0.75	0.2319

### Latent class profiles

**Fig. 1:**
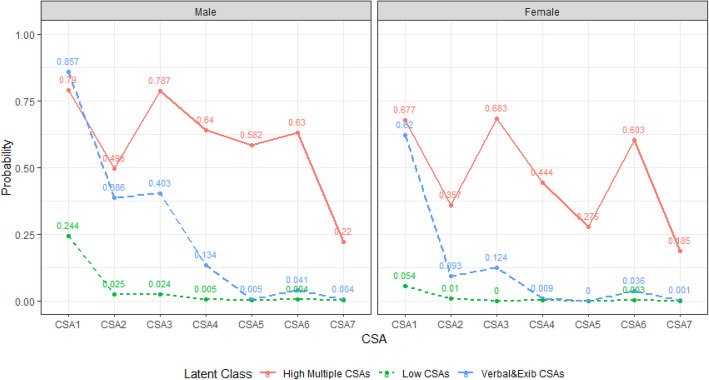
Profile probabilities of CSA latent class analysis

As shown in Fig. 1, the three profiles of CSAs were characterized by the following conditional probabilities: In “high multiple CSAs”, there was a high probability of positive response on each CSA item. In “Verbal or exhibitionism CSA”, a higher probability of positive response was seen on the item “Having been told dirty jokes or shown pornographic pictures, publications or supplies. while lower probability on items such as “Having had someone rubbing his/her genitals on you”, “Having had someone touching your genitals or forcing you to make contact with his/her genitals by mouth”, “Having had someone attempting to have sex with you” and “Having had someone forcing to have sex with you”. In “Low CSAs”, there was a low probability of positive response on each topic. Among them, the “Low CSAs” accounted for the largest proportion (boys 96.1%, girls 95.2%). A smaller proportion of respondents was classified as the “Verbal or exhibitionism CSA”(boys 2.6%, girls 3.9%). The number of respondents in the “high multiple CSAs” is the smallest. The proportion of “high multiple CSAs” in boys is higher than that in girls (1.3% vs. 0.9%).

**Fig. 2:**
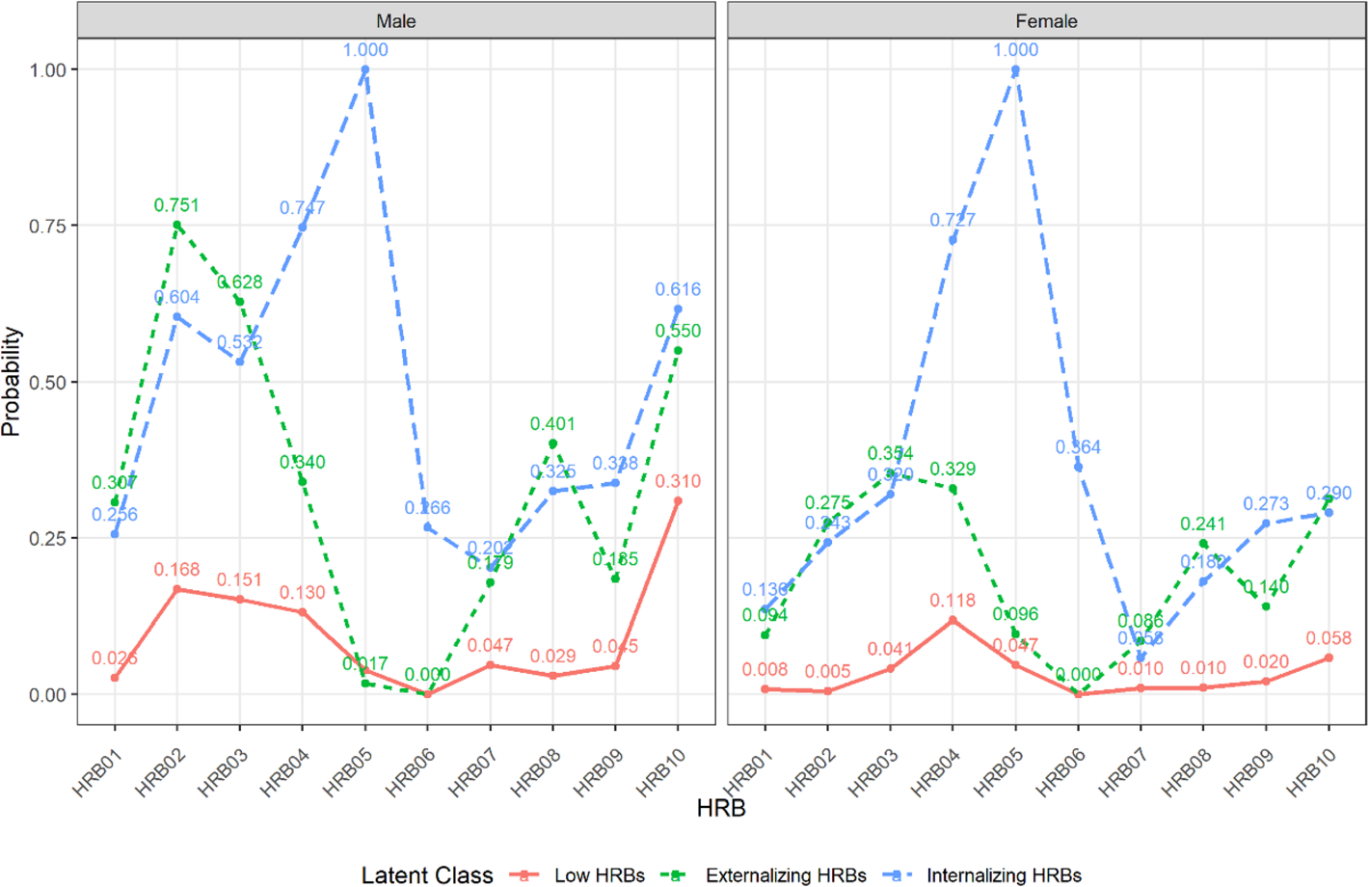
Profile probability of HRB latent class analysis

As shown in Fig. 2, the three profiles of HRBs were characterized by the following probabilities: The “Low HRBs”, which accounted for the largest proportion (70.5%) in the population, had the lowest probability of positive response on each HRB item. A smaller proportion (20.7%) of the population belonged to “externalizing HRBs” which showed a higher probability of positive response on topics such as “fondling “, “smoking”, “drinking alcohol”, “gambling”, “skipping classes” and “fighting”, while a lower probability of positive response on “depression, despair, or extreme anxiety”, “suicidal ideation”, and “suicidal behavior”. The lowest proportion in the population of this latent class was taken up by “internalizing HRBs”(8.7%), which had a higher probability of positive responses on topics such as “depression, despair, or extreme anxiety”, “suicidal ideation” and “suicidal behavior”, while a lower probability of positive responses on the remaining topics. Compared between genders, girls were more prominent in internalization behaviors, while boys presented a two-peak distribution of both internalization and externalization of the latent conditional probabilities.

### Association analysis

In Table 5, results of the dual LCA regression model among boys showed that: considering “Low HRBs” as the baseline, there was a significant association between “externalizing HRBs” and “Verbal or exhibitionism CSA” (OR 2.51, 95% CI 1.50–4.20), and a significant association between the “externalizing HRBs” and “high multiple CSAs” (OR 4.05, 95% CI 1.71–9.57). There was also a significant association between “Internalizing behaviors “ and “Verbal or exhibitionism CSA” (OR 3.08, 95% CI 1.48–6.40); and a significant association between the “ Internalizing behaviors “ and “high multiple CSAs” (OR11.77, 95% CI 4.76–29.13). The association of “Verbal or exhibitionism CSA” and “high multiple CSAs” in girls was of greater strength, which almost doubled that in boys (male OR 3.08, 95% CI 1.48–6.40 vs female OR 6.05, 95% CI 3.73–9.80). After adjusted by variables such as school, family, and personal lifestyle, the above associations still existed, and their directions remained the same, with little difference in strength.

**Table 5:** Association between CSA classes and HRB classes

	HRB Latent Classes							
	Proportion				Crude Association		Adjusted Association ^a^	
CSA Latent classes	Total	Low HRBs	Externalizing HRBs	Internalizing HRBs	Externalizing HRBs	Internalizing HRBs	Externalizing HRBs	Internalizing HRBs
	N (%)	N (%)	N (%)	N (%)	OR[95%CI]	OR[95%CI]	OR[95%CI]	OR[95%CI]
Male								
Low CSAs	4170 (96.1)	2912 (97.9)	969 (93.1)	289* (88.9)	REF	REF	REF	REF
Verbal or exhibitionism CSAs	114 (2.6)	50 (1.7)	49 (4.7)	15 (4.6)	2.95 [1.97–4.40]	3.02 [1.68–5.45]	2.51 [1.50–4.20]	3.08 [1.48–6.40]
High multiple CSAs	55 (1.3)	11 (0.4)	23 (2.2)	21 (6.5)	6.28 [3.05–12.94]	19.24 [9.18–40.29]	4.05 [1.71–9.57]	11.77 [4.76–29.13]
Total	4339	2973	1041	325				
Female								
Low CSAs	4196 (95.2)	3118 (97.6)	700 (90.6)	378* (85.9)	REF	REF	REF	REF
Verbal or exhibitionism CSAs	171 (3.9)	67 (2.1)	57 (7.4)	47 (10.7)	3.79 [2.64–5.45]	5.79 [3.93–8.53]	2.53 [1.63–3.95]	6.05 [3.73–9.80]
High multiple CSAs	40 (0.9)	9 (0.3)	16 (2.1)	15 (3.4)	7.92 [3.48–17.99]	13.75 [5.98–31.63]	4.97 [1.99–12.44]	9.87 [3.71–26.25]
Total	4407	3194	773	440				

^a^Adjusted Association: Adjusted for variables associated with school, family function, and respondents’ individual free time lifestyle

## Discussion

Across the globe, the self-report prevalence of CSA varies greatly among different studies ranging from 2 to 62% [4–6], and the average is estimated to be 12% [4]. According to a meta-analysis that synthesized CSA prevalence of Chinese studies [39], prevalence of CSA showed large variation from 2%(Yen, 2008)to 35.2%(Zhao&Li, 2006)of self-reported at least one form of CSA experiences in China [41]. Different studies have used different CSA items to define CSA, which largely affects the estimated prevalence [42]. Estimates from broad definitions of CSA that includes non-contact CSA yield higher prevalence compared with estimates from narrow definition of CSA that merely include contact CSA (Chen et al., 2010). According to Ji’s review about CSA studies in China, the prevalence of contact CSA varied from 3.3% (Ye, Tao, Fang, Huang, & Sun, 2006) to 14.5% (Chen, Dunne, & Han, 2004), and that of penetrative CSA varied from 0.4% (Chen, Dunne, et al., 2004; Li, 2008) to 2.9% (Chen, Dunne, & Han, 2006) in females. The prevalence of contact CSA varied from 3% (So-kum Tang, 2002) to 15% (Chen, Wang, & Dunne, 2003), and that of penetrative CSA varied from 0 (Li, 2008) to 2.9% (Xia et al., 2009) in males. Our research has produced consistent results. The prevalence of experiencing at least one form of CSAs is 12.9% among the participated students in China. The prevalence of each measured sexual abuse item varies from 1.0 to 4.4%. Non-contact CSAs are the most common forms. Some current studies have shown that the majority of victims were under 14 years old when they encountered CSA [43], and the least age difference between the perpetrator and the victim was 5 years [44]; while in some other studies, the age cut-off point is set as 16 years old [45–47] or 18 years old [48, 49]. The discrepancy in study population is also one of the reasons for the difference, as the research objects in Sun’s research are adults [48], while those in Chen’s research [50] are college students in selected areas. Variations of prevalence from different studies are mainly due to different definition of CSA experiences, different constrains to the age of the victim/the perpetrator, different situations in which CSA occurs, and different recruitments of respondents as well. The direct comparison should be done with caution.

Traditional studies recommended CSA to be classified into four types, namely (1) non-contact, (2) genital touching, (3) attempted vaginal or anal insertion, and (4) vaginal or anal penetration. Empirical opinions showed that CSA involving types (2), (3), and (4) are more likely to have significant negative consequences. Other studies suggested to group CSA into two types, one being body-contact CSA and the other being non-contact CSA. However, in real life, victims often encounter more than one type of CSA [51]. Traditional classification methods set empirical cut-off values for classification, dividing children into mutually exclusive categories. One advantage of these methods is that the classification is easy to create and compare. The disadvantage is that they required priori assumptions about the adverse impact, and often took into account only one type of the most serious sexual abuse experience that an individual had experienced. They cannot extract how and to what extent various types of abuse coexist in the target population. Thus, some other studies tried cluster analysis to explore more reasonable CSA classification. For example, a study conducted on 303 teenagers in Denmark resulted in a model of four latent classes, namely, multiple CSA latent class, high language/low contact latent class, high sexual contact latent class and nonvictim latent class [52].

The results of our study are consistent with the literatures highlighting subgroups of CSA by latent class analysis rather than the traditional empirical approach. Compared with the categories obtained according to the traditional classification, the latent class of CSA obtained in our study has taken into account both heterogeneity and co-occurrence. Based on the conditional probability and local independence, this study obtained a three-class latent variable to characterize the latent categorical profiles behind CSA experience as “Low CSAs “, “Verbal or exhibitionism CSA”, and “High Multiple CSAs”. The results of our study and the Danish study found similar latent class subgroups of CSA victims, characterized by high language/low contact latent class, high multiple CSA class and low CSA latent class, which reflected that the LCA method is helpful and stable in detecting latent CSA subgroups. In another study trying using LCA, a sample of 657 young people recruited from high schools and colleges was examined to identify latent class of sexually abusive perpetrators [53]. In this analysis, latent classes of kissing/caressing, attempting sexual intercourse, and completed sexual intercourse were established based on CSA items, which were then found to be related to the abusive behaviors of verbal coercion, material seduction, and violent coercion of the perpetrators. It can be seen that LCA can help to find homogeneous subgroups and the association between the groups and some certain distal variables.

Our study also fits a three-category latent class variable according to the heterogeneity of HRBs. The result is consistent with the results of some other researches. Zlotnick [54] believed that some characteristics of complex posttraumatic stress disorder(PTSD) should coexist with internalized indicators (for example, ineffectiveness, shame, depression/ despair, social withdrawal, and physical discomfort), while other characteristics should be more related to externalization indicators (for example, self-destructive behavior, impulsive actions, and hostility). Miller’s research [55] indicated internalization and externalization show higher scores on complex PTSD rather than simple PTSD in a series of scales measuring the core concept of PTSD. These findings highlight the heterogeneous of patients with complex adverse experiences. The practice of the LCA method in these researches suggests that the latent category method can better take into account the consistency within group and the difference between groups. The attempts of these studies using the LCA method suggest that the LCA method has great potential in the field of exploring adolescent behavior co-occurrence and heterogeneity.

Studies have suggested that the effects of CSA are cumulative. Exposure to high levels or multiple forms of CSA experiences may have more harmful and more reversible effects [23]. In our study, the results of association analysis showed that, adolescents presented diverse patterns of HRBs according to different patterns of CSA experience. Compared to the students classified as “Low HRBs”, those classified as “externalizing HRBs” were associated with a high probability of “Verbal or exhibitionism CSA”, and a similar association was evident among those in “internalizing HRBs”. Meanwhile, compared with “Low CSAs”, those in “high multiple CSAs”, have the strongest association with both “externalizing HRBs” and “internalizing HRBs”. All these associated directions were positive and statistically significant, which are consistent with the research hypothesis. Existing studies suggest that children with CSA experiences show higher risk of engaging in health-risk behaviors, such as drinking, smoking, gambling, and suicidal ideation [38, 49, 56, 57], or being less ambitious, having fewer friends, and having lower self-esteem [10, 58], and then their lives may have been traumatically disrupted by these behaviors [12–16, 59]. Compared with those who have received low or no abuse, multiple victimization are associated with an increased risk of externalization problems [60] or internalizing problems [61–65]. Our results not only confirmed the relation between CSA and risk behaviors, but also showed a dose-response association between the amount of ACE and behavioral and health issues, which were consistent with some of the few current studies, which means multiple victims are more likely to have behavioral or psychological problems. Sexual assault or forced penetrated abuse is more likely to associate with more negative long-term consequences [66–68] compared with non-forced or non-penetrated sexual abuse. More severe forms of sexual abuse are more destructive because they reinforce feelings of helplessness, powerlessness and self-blame [69]. Our study supports previous studies that multiple CSA experiences may lead to a decrease in the overall perception of one’s health [70] and an increase in negative health behaviors [71]. Abuse prevalence are believed to undermine the ability of adolescents to complete key developmental tasks, promoting them to use ineffective or even harmful coping strategies. The difference in population patterns can help researchers understand the homogeneity and heterogeneity between individuals, so as to provide a basis for the design and implementation of targeted intervention measures for different subtypes of victimization.

Previous studies showed that boys and girls who experienced CSA may have similar physical and mental health sequelae [24, 72, 73]. Our study showed that some of the association strength differed between genders. The associations were nearly twice as strong in girls as it was in boys (male OR = 3.08, 95%CI 1.48–6.40 vs female OR = 6.05, 95%CI 3.73–9.80). The results suggest that boys and girls may differ in how they respond to negative events [74, 75]. Females tend to internalize stress into shyness, shame, guilt, sadness, and self-hostility [76], while males are more likely to respond with externalizing behaviors such as problematic alcohol use or committing violent acts [77]. However, there are controversies in existing studies on the impact of gender on the associations of CSA and HRB. Coohey [78] suggested that sexually abused boys were more likely than girls to have an internalizing behavior problem using a clinical sample, while Maikovich-Fong [61, 72] claimed that sex did not moderate the relation between abuse characteristics and youth emotional and behavioral problems. These findings highlight the importance of gender in the field of CSA research.

### Limitations

This study has common limitations of self-reported retrospective research. The possibility of underreport or underestimation cannot be ruled out. Due to the strict sampling frame and the high response rate of this study, volunteer bias should be minimal. Different studies have used different CSA items to define CSA, which largely affects the estimated prevalence [42]. The items of CSA experience measured in this study was embedded in a large national study. Due to the limitation of the investigation duration, the simplified scale adapted based on existing CSA measurements was not a generally accepted scale. It is difficult to avoid the impact of measurement bias and recall bias using self-reporting to collect data, which limit future comparisons between our results and other studies using different measurements. The cross-sectional design of our study was not capable of capturing chronological or cause-sequence order. The respondents were between 10 and 18 years old and had not yet experienced the full adolescence, so many of the abuse experiences or health risk behaviors will not yet have crystallized. Our findings about school students may not be extended to out-of-school children or CSA from peers. For these subpopulations, CSA prevalence or patterns may be different. Therefore, these are also possible causes of CSA underestimation. Application of LCA enables us to identify and categorize the target population into heterogeneity subgroups. The misclassification assigned to each latent class is reasonable and the probabilities of classification error were fairly accepted. However, uncertainty [79] in model class membership still exists. The results should be cautiously interpreted. Rare prevalence of CSA experiences, underestimation and potential misclassification may cause smaller sample cells and may reduce the statistical power. Due to the covert and sensitivity nature of the survey contents, randomization, restrictions or matching were not applied to controlled potential confounding, while we adopted gender stratification and multivariate dual LCA regression model to controll measured confounders, in order to reduce potential bias. Our analysis was based on a limited number of covariates, thus revealing a broader range of confounding factors that may also influence the association between CSA and HRB is in need. Further considerations should be tested in cooperating broader potential mediators, moderators and their possible impacts on adolescent adverse experiences or behaviors.

## Conclusion and implication

Notwithstanding the limitations, our study contributes to the scarce literature on exploring prevalence and co-occurrence subgroups of Chinese school teens reporting sexual abuse and examines the association between CSA and HRB patterns. The prevalence of experiencing at least one form of CSAs is 12.9%. Non-contact CSAs are the most common forms. The co-occurrence and heterogeneity of the experiences play a role in the association of CSA and HRB. Students who have experienced multiple-victimization of CSA have a higher probability of HRB risk, especially “depression, despair, or extreme anxiety”, “suicidal ideation” and “suicidal behavior”. The findings emphasize the importance of considering heterogeneity and co-occurrence in studies of adverse childhood experiences. The analysis could improve understandings of the potential vulnerabilities. Although contact or non-contact CSA are considered public health problems, multi-victimization has more significantly negative effects. Children with multiple abuse experiences should be paid more attention to for risk behavior interventions. The results could provide new evidence and research directions for association between CSA and HRB, and help revise the interventions for different subgroups of victims.

### Suggestions for future studies

This study aims to explore the potential mechanism and to provide new clues and research directions for the association between CSA and HRB. Future research that replicates this research is needed to generalize these findings to similar or different samples of adolescents, and to examine differences between subgroups with specific risk factors that have the potential to mediate or moderate the association, which could help develop more nuanced interventions addressing adverse experiences and risk behaviors among at-risk adolescents.

## Supplementary Information

**Additional file 1: ** The table shows CSA prevalence stratified by the participant demographic characteristics.

## Data Availability

The datasets used and/or analyzed during the current study are available from the corresponding author on reasonable request.

## Abbreviations

CSA: Child sexual abuse
HRB: Health risk behaviors
OR: Odds Ratio
CI: Confidence Interval
LCA: Latent class analysis
ISPCAN: International Society for Prevention of Child Abuse and Neglect
AIC: Akaike Information Criterion
BIC: Bayesian Information Criterion
aBIC: Adjusted Bayesian Information Criterion
