# Supplementary material for: Co-occurrence subgroups of child sexual abuse, health risk behaviors and their associations among secondary school students in China
Source: BMC Public Health. 2021 Jun 14;21:1139. doi: 10.1186/s12889-021-11199-5 (PMC8201738; doi:10.1186/s12889-021-11199-5)
Supplement: Supplementary file 1 — Additional file 1: The table shows CSA prevalence stratified by the participant demographic characteristics. [file 12889_2021_11199_MOESM1_ESM.docx]

Additional Table 1 CSA prevalence stratified by the participant demographic characteristics

|  |  | Sample Size | Any CSA | | CSA1 | | CSA2 | |  | CSA3 | |  | CSA4 | | CSA5 | |  | CSA6 | | CSA7 | |
| --- | --- | --- | --- | --- | --- | --- | --- | --- | --- | --- | --- | --- | --- | --- | --- | --- | --- | --- | --- | --- | --- |
|  |  | n | n | % | n | % | n | % |  | n | % |  | n | % | n | % |  | n | % | n | % |
| Grade | Seventh grade | 2246 | 241 | 10.73 * | 69 | 3.07 * | 115 | 5.12 |  | 57 | 2.54 * |  | 31 | 1.38 * | 19 | 0.85 * |  | 26 | 1.16 * | 18 | 0.80 * |
|  | Eighth grade | 1147 | 124 | 10.81 | 53 | 4.62 | 48 | 4.18 |  | 34 | 2.96 |  | 17 | 1.48 | 11 | 0.96 |  | 18 | 1.57 | 6 | 0.52 |
|  | Ninth grade | 1021 | 134 | 13.12 | 56 | 5.48 | 39 | 3.82 |  | 44 | 4.31 |  | 12 | 1.18 | 7 | 0.69 |  | 15 | 1.47 | 6 | 0.59 |
|  | Tenth grade | 2431 | 375 | 15.43 | 122 | 5.02 | 114 | 4.69 |  | 119 | 4.9 |  | 60 | 2.47 | 42 | 1.73 |  | 87 | 3.58 | 36 | 1.48 |
|  | Eleventh grade | 1092 | 139 | 12.73 | 46 | 4.21 | 38 | 3.48 |  | 48 | 4.4 |  | 22 | 2.01 | 14 | 1.28 |  | 38 | 3.48 | 14 | 1.28 |
|  | Twelfth grade | 809 | 118 | 14.59 | 36 | 4.45 | 35 | 4.33 |  | 50 | 6.18 |  | 24 | 2.97 | 9 | 1.11 |  | 27 | 3.34 | 5 | 0.62 |
| Gender | Male | 4339 | 613 | 14.13 * | 234 | 5.39 * | 208 | 4.79 |  | 184 | 4.24 |  | 100 | 2.30 * | 64 | 1.47 * |  | 88 | 2.03 * | 39 | 0.9 |
|  | Female | 4407 | 518 | 11.75 | 148 | 3.36 | 181 | 4.11 |  | 168 | 3.81 |  | 66 | 1.5 | 38 | 0.86 |  | 123 | 2.79 | 46 | 1.04 |
| Area | Urban | 4305 | 520 | 12.08 * | 192 | 4.46 | 180 | 4.18 |  | 156 | 3.62 |  | 75 | 1.74 | 57 | 1.32 |  | 104 | 2.42 | 40 | 0.93 |
|  | Rural | 4441 | 611 | 13.76 | 190 | 4.28 | 209 | 4.71 |  | 196 | 4.41 |  | 91 | 2.05 | 45 | 1.01 |  | 107 | 2.41 | 45 | 1.01 |
| Whether to live on campus | No | 4404 | 520 | 11.81 * | 180 | 4.09 | 194 | 4.41 |  | 161 | 3.66 |  | 73 | 1.66 | 48 | 1.09 |  | 90 | 2.04 * | 32 | 0.73 * |
|  | Yes | 4342 | 611 | 14.07 | 202 | 4.65 | 195 | 4.49 |  | 191 | 4.4 |  | 93 | 2.14 | 54 | 1.24 |  | 121 | 2.79 | 53 | 1.22 |
| Academic performance | Good | 3605 | 413 | 11.46 * | 126 | 3.50 * | 146 | 4.05 * |  | 135 | 3.74 |  | 49 | 1.36 * | 34 | 0.94 * |  | 74 | 2.05 * | 25 | 0.69 * |
|  | Fair | 3602 | 473 | 13.13 | 156 | 4.33 | 157 | 4.36 |  | 142 | 3.94 |  | 76 | 2.11 | 37 | 1.03 |  | 84 | 2.33 | 34 | 0.94 |
|  | Poor | 1539 | 245 | 15.92 | 100 | 6.5 | 86 | 5.59 |  | 75 | 4.87 |  | 41 | 2.66 | 31 | 2.01 |  | 53 | 3.44 | 26 | 1.69 |
| Peer relationship | Good | 5255 | 631 | 12.01 * | 210 | 4.00 * | 202 | 3.84 * |  | 201 | 3.82 * |  | 99 | 1.88 | 68 | 1.29 * |  | 123 | 2.34 * | 38 | 0.72 * |
|  | Fair | 3266 | 459 | 14.05 | 156 | 4.78 | 168 | 5.14 |  | 135 | 4.13 |  | 60 | 1.84 | 28 | 0.86 |  | 77 | 2.36 | 40 | 1.22 |
|  | Poor | 225 | 41 | 18.22 | 16 | 7.11 | 19 | 8.44 |  | 16 | 7.11 |  | 7 | 3.11 | 6 | 2.67 |  | 11 | 4.89 | 7 | 3.11 |
| Sibling number | None | 4350 | 533 | 12.25 * | 178 | 4.09 | 182 | 4.18 |  | 186 | 4.28 * |  | 68 | 1.56 * | 48 | 1.1 |  | 99 | 2.28 * | 32 | 0.74 * |
|  | One | 3165 | 402 | 12.7 | 142 | 4.49 | 145 | 4.58 |  | 105 | 3.32 |  | 61 | 1.93 | 39 | 1.23 |  | 61 | 1.93 | 31 | 0.98 |
|  | Two or more | 1231 | 196 | 15.92 | 62 | 5.04 | 62 | 5.04 |  | 61 | 4.96 |  | 37 | 3.01 | 15 | 1.22 |  | 51 | 4.14 | 22 | 1.79 |
| Marital status of parents | Normal / married | 7782 | 968 | 12.44 * | 337 | 4.33 | 328 | 4.21 * |  | 304 | 3.91 |  | 142 | 1.82 | 79 | 1.02 * |  | 173 | 2.22 * | 63 | 0.81 * |
|  | Divorced / separation | 670 | 103 | 15.37 | 25 | 3.73 | 45 | 6.72 |  | 27 | 4.03 |  | 10 | 1.49 | 14 | 2.09 |  | 26 | 3.88 | 12 | 1.79 |
|  | Death of one or both parents | 233 | 45 | 19.31 | 14 | 6.01 | 11 | 4.72 |  | 16 | 6.87 |  | 9 | 3.86 | 5 | 2.15 |  | 9 | 3.86 | 6 | 2.58 |
| Parents’ relationship | Harmonious | 5189 | 584 | 11.25 * | 206 | 3.97 * | 211 | 4.07 |  | 168 | 3.24 * |  | 85 | 1.64 | 47 | 0.91 |  | 101 | 1.95 * | 46 | 0.89 |
|  | Not harmonious | 2593 | 384 | 14.81 | 131 | 5.05 | 117 | 4.51 |  | 136 | 5.24 |  | 57 | 2.2 | 32 | 1.23 |  | 72 | 2.78 | 17 | 0.66 |
| Father's education | Elementary and below | 1298 | 219 | 16.87 * | 65 | 5.01 | 81 | 6.24 * |  | 74 | 5.70 * |  | 35 | 2.7 | 21 | 1.62 |  | 36 | 2.77 | 22 | 1.69 * |
|  | Junior high school | 4276 | 535 | 12.51 | 185 | 4.33 | 178 | 4.16 |  | 168 | 3.93 |  | 78 | 1.82 | 42 | 0.98 |  | 106 | 2.48 | 33 | 0.77 |
|  | High school / Vocational school | 2002 | 228 | 11.39 | 80 | 4 | 76 | 3.8 |  | 69 | 3.45 |  | 29 | 1.45 | 21 | 1.05 |  | 38 | 1.9 | 13 | 0.65 |
|  | College and above | 670 | 80 | 11.94 | 26 | 3.88 | 30 | 4.48 |  | 19 | 2.84 |  | 15 | 2.24 | 9 | 1.34 |  | 17 | 2.54 | 5 | 0.75 |
| Relationship with father | Good | 5117 | 581 | 11.35 * | 203 | 3.97 * | 215 | 4.20 * |  | 167 | 3.26 * |  | 78 | 1.52 * | 46 | 0.90 * |  | 99 | 1.93 * | 42 | 0.82 * |
|  | Fair | 2283 | 318 | 13.93 | 105 | 4.6 | 93 | 4.07 |  | 105 | 4.6 |  | 52 | 2.28 | 31 | 1.36 |  | 64 | 2.8 | 19 | 0.83 |
|  | Poor | 1346 | 232 | 17.24 | 74 | 5.5 | 81 | 6.02 |  | 80 | 5.94 |  | 36 | 2.67 | 25 | 1.86 |  | 48 | 3.57 | 24 | 1.78 |
| Severity of father | Strict | 1199 | 139 | 11.59 * | 50 | 4.17 * | 52 | 4.34 * |  | 39 | 3.25 * |  | 20 | 1.67 * | 15 | 1.25 * |  | 31 | 2.59 * | 14 | 1.17 |
|  | Mostly strict | 3850 | 436 | 11.32 | 155 | 4.03 | 150 | 3.9 |  | 129 | 3.35 |  | 60 | 1.56 | 32 | 0.83 |  | 64 | 1.66 | 28 | 0.73 |
|  | Mostly easy-going | 2660 | 364 | 13.68 | 111 | 4.17 | 117 | 4.4 |  | 119 | 4.47 |  | 55 | 2.07 | 33 | 1.24 |  | 71 | 2.67 | 26 | 0.98 |
|  | Easy-going | 1037 | 192 | 18.51 | 66 | 6.36 | 70 | 6.75 |  | 65 | 6.27 |  | 31 | 2.99 | 22 | 2.12 |  | 45 | 4.34 | 17 | 1.64 |
| Mother's education | Elementary and below | 1244 | 207 | 16.64 * | 59 | 4.74 | 75 | 6.03 * |  | 71 | 5.71 * |  | 34 | 2.73 | 19 | 1.53 |  | 34 | 2.73 | 21 | 1.69 * |
|  | Junior high school | 4092 | 508 | 12.41 | 176 | 4.3 | 168 | 4.11 |  | 160 | 3.91 |  | 76 | 1.86 | 40 | 0.98 |  | 98 | 2.39 | 29 | 0.71 |
|  | High school / Vocational school | 1810 | 199 | 10.99 | 72 | 3.98 | 66 | 3.65 |  | 59 | 3.26 |  | 25 | 1.38 | 17 | 0.94 |  | 34 | 1.88 | 12 | 0.66 |
|  | College and above | 1600 | 217 | 13.56 | 75 | 4.69 | 80 | 5 |  | 62 | 3.88 |  | 31 | 1.94 | 26 | 1.63 |  | 45 | 2.81 | 23 | 1.44 |
| Relationship with mother | Good | 5926 | 700 | 11.81 * | 245 | 4.13 | 235 | 3.97 * |  | 205 | 3.46 * |  | 110 | 1.86 | 69 | 1.16 |  | 131 | 2.21 * | 55 | 0.93 * |
|  | Fair | 1949 | 271 | 13.9 | 90 | 4.62 | 89 | 4.57 |  | 89 | 4.57 |  | 39 | 2 | 21 | 1.08 |  | 48 | 2.46 | 12 | 0.62 |
|  | Poor | 871 | 160 | 18.37 | 47 | 5.4 | 65 | 7.46 |  | 58 | 6.66 |  | 17 | 1.95 | 12 | 1.38 |  | 32 | 3.67 | 18 | 2.07 |
| Severity of mother | Strict | 1771 | 214 | 12.08 * | 81 | 4.57 | 74 | 4.18 * |  | 69 | 3.90 * |  | 35 | 1.98 | 25 | 1.41 |  | 48 | 2.71 * | 18 | 1.02 |
|  | Mostly strict | 3842 | 445 | 11.58 | 149 | 3.88 | 138 | 3.59 |  | 132 | 3.44 |  | 75 | 1.95 | 36 | 0.94 |  | 72 | 1.87 | 29 | 0.75 |
|  | Mostly easy-going | 2528 | 358 | 14.16 | 117 | 4.63 | 132 | 5.22 |  | 112 | 4.43 |  | 42 | 1.66 | 30 | 1.19 |  | 65 | 2.57 | 31 | 1.23 |
|  | Easy-going | 605 | 114 | 18.84 | 35 | 5.79 | 45 | 7.44 |  | 39 | 6.45 |  | 14 | 2.31 | 11 | 1.82 |  | 26 | 4.3 | 7 | 1.16 |
| Total |  | 8746 | 1131 | 12.93 | 382 | 4.37 | 389 | 4.45 |  | 352 | 4.02 |  | 166 | 1.9 | 102 | 1.17 |  | 211 | 2.41 | 85 | 0.97 |

*：p<0.05
